# DELFOS—drug efficacy leveraging forked and specialized networks—benchmarking scRNA-seq data in multi-omics-based prediction of cancer sensitivity

**DOI:** 10.1093/bioinformatics/btad645

**Published:** 2023-10-20

**Authors:** Luiz Felipe Piochi, António J Preto, Irina S Moreira

**Affiliations:** Department of Life Sciences, University of Coimbra, Coimbra 3000-456, Portugal; CNC—Center for Neuroscience and Cell Biology, Center for Innovative Biomedicine and Biotechnology, University of Coimbra, Coimbra, Portugal; CIBB—Center for Innovative Biomedicine and Biotechnology, Coimbra 3004-504, Portugal; CNC—Center for Neuroscience and Cell Biology, Center for Innovative Biomedicine and Biotechnology, University of Coimbra, Coimbra, Portugal; CIBB—Center for Innovative Biomedicine and Biotechnology, Coimbra 3004-504, Portugal; PhD Programme in Experimental Biology and Biomedicine, Institute for Interdisciplinary Research (IIIUC), University of Coimbra, Coimbra 3030-789, Portugal; Department of Life Sciences, University of Coimbra, Coimbra 3000-456, Portugal; CNC—Center for Neuroscience and Cell Biology, Center for Innovative Biomedicine and Biotechnology, University of Coimbra, Coimbra, Portugal; CIBB—Center for Innovative Biomedicine and Biotechnology, Coimbra 3004-504, Portugal

## Abstract

**Motivation:**

Cancer is currently one of the most notorious diseases, with over 1 million deaths in the European Union alone in 2022. As each tumor can be composed of diverse cell types with distinct genotypes, cancer cells can acquire resistance to different compounds. Moreover, anticancer drugs can display severe side effects, compromising patient well-being. Therefore, novel strategies for identifying the optimal set of compounds to treat each tumor have become an important research topic in recent decades.

**Results:**

To address this challenge, we developed a novel drug response prediction algorithm called Drug Efficacy Leveraging Forked and Specialized networks (DELFOS). Our model learns from multi-omics data from over 65 cancer cell lines, as well as structural data from over 200 compounds, for the prediction of drug sensitivity. We also evaluated the benefits of incorporating single-cell expression data to predict drug response. DELFOS was validated using datasets with unseen cell lines or drugs and compared with other state-of-the-art algorithms, achieving a high prediction performance on several correlation and error metrics. Overall, DELFOS can effectively leverage multi-omics data for the prediction of drug responses in thousands of drug–cell line pairs.

**Availability and implementation:**

The DELFOS pipeline and associated data are available at github.com/MoreiraLAB/delfos.

## Graphical Abstract

**Graphical Abstract:**
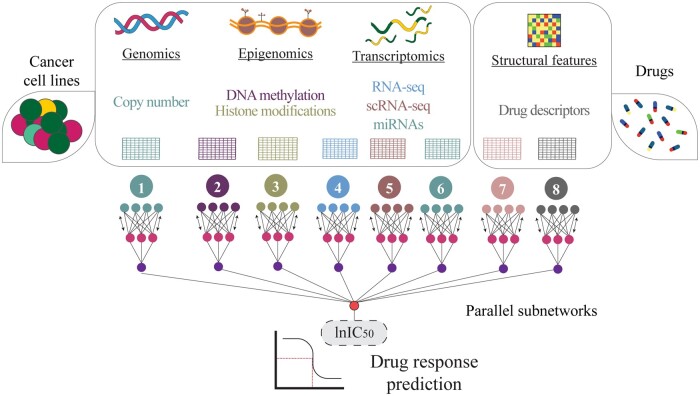

## 1 Introduction

Tumors are highly plastic and heterogeneous entities composed of many different cell types, with distinct expression and metabolic profiles. Consequently, tumor plasticity is linked to drug resistance, as some cancer cells may survive, divide, and give rise to a resistant cell population, ultimately leading to disease recurrence. The average discovery and development process is estimated to take over a decade and $2 billion for each drug (Hinkson *et al.* 2020). Thus, computational approaches and Artificial Intelligence technology have recently received much attention as strategies to address these limitations and are expected to expand even more in the following years (Mao *et al.* 2021, Sun *et al.* 2022).

Omics data have been relevant for several research areas in addition to disease biology, from nutrition to systems biology, and their applications have greatly expanded in recent decades owing to technological advances and improved computational tools (Misra *et al.* 2018). More recently, as the technology required to generate this type of data has become more accessible, research has shifted its focus to the integrated analyses of different omics modalities. Together, each omics type contributes to the depiction of the cellular environment, similar to each color contributing to the final image of the kaleidoscope. Therefore, multimodal systematic analyses of drug susceptibility in various tissues can be a significant step toward better treatment outcomes in most disease scenarios, from neurodegeneration (Zhou *et al.* 2020, Xu *et al.* 2021) to cancer (McFarland *et al.* 2020, Aissa *et al.* 2021). Although bulk sequencing methods can provide a general perspective on drug effects in a tissue, responses generated by larger cell subpopulations may hide the impact on other groups of cells (Trapnell 2015, Srivatsan *et al.* 2020). Fortunately, technological advances have led to a paradigm shift in omics research, as scientists can now obtain information at single-cell resolution, such as Single-Cell RNA-Sequencing (scRNA-Seq) data, to address these shortcomings.

In recent decades, many types of cancer phenotypic datasets have been created, such as the Cancer Cell Line Encyclopedia (CCLE) (Barretina *et al.* 2012) and the Genomics of Drug Sensitivity in Cancer (GDSC) (Yang *et al.* 2013). As these and other large portals and databases of cellular and pharmacogenomic data have become available, many Machine Learning (ML) algorithms have been developed in recent years for drug response prediction (DRP) based on phenotypic cellular data (Chen and Zhang 2021, Firoozbakht *et al.* 2022). These algorithms allow automated learning from pharmacological data, and then become able to predict, with varying inputs, omics types, targets, and downstream applications, how cells will likely respond to different drugs (Adam *et al.* 2020). In addition to phenotypic information, drug descriptors or molecular fingerprints that define the structural features of drugs have become common tools for building these models (Bazgir *et al.* 2020, Choi *et al.* 2020, Liu *et al.* 2020, Chawla *et al.* 2022, Firoozbakht *et al.* 2022). Thus, recent models have elaborated on conjugating biological data with drug structural data to predict drug sensitivities.

Given the large amounts of different types of data available and the need for better computational tools to assist in drug development, we developed a novel Deep Learning (DL) model for drug sensitivity prediction in cancer cell lines, named Drug Efficacy Leveraging Forked Optimized and Specialized networks (DELFOS). Specifically, our algorithm can be used to predict the ln(IC_50_) of drug–cell line combinations by learning from different omics types from cancer cell lines and the structural features of drugs. We also evaluated the influence of using scRNA-seq data, in addition to other data types, on the prediction performance of DELFOS.

## 2 Materials and methods

### 2.1 scRNA-seq datasets

All scRNA-seq data were retrieved from publicly available datasets. Five scRNA-seq datasets describing gene expression in different cancer cell lines treated with 0.1%–0.2% DMSO were collected (Table 1). All data were downloaded from the Gene Expression Omnibus (https://www.ncbi.nlm.nih.gov/geo/), except for the data from McFarland *et al.* (2020), which were retrieved from Figshare (https://figshare.com/s/139f64b495dea9d88c70).

**Table 1.** Summary of selected scRNA-Seq datasets.

Reference	Cancer type	Platform	Identifier
(Sriramkumar *et al.* 2022)	Ovarian	Chromium	GSE207993
(McFarland *et al.* 2020)	Several	MIXseq	figshare.com/s/139f64b495dea9d88c70
(Schnepp *et al.* 2020)	Prostate	C1	GSE140440
(Kagohara *et al.* 2020)	HNSCC	Chromium	GSE137524
(Ben-David *et al.* 2018)	Breast	Chromium	GSE114462

All scRNA-seq data were annotated and processed using Seurat v3 R library (Butler *et al.* 2018). First, all datasets were filtered such that only the genes shared between all datasets were selected. Next, Seurat objects for each dataset were created using only cells expressing at least 1000 genes and filtered by the percentage of expressed mitochondrial genes to remove low-quality cells. Accordingly, cells expressing over 15% of mitochondrial genes were filtered out (Supplementary Figs S1–S5). Datasets were then merged using genes in common and normalized according to standard Seurat parameters. The 2000 genes with the highest variability in the merged dataset were selected for further processing. Cell cycle effects were scored according to the list of G2/M and S phase genes by Tirosh *et al.* (2016) (Supplementary Fig. S6). Finally, the data were scaled and centered using Seurat standard parameters.

### 2.2 Bulk omics datasets

Five datasets of different omics data from several cancer cell lines were retrieved from the CCLE 2019 and DepMap Public 22Q2 public releases in the DepMap Portal (https://depmap.org/portal/ccle/), and included protein-coding gene expression, copy number variation, chromatin profiling, miRNA expression, and methylation data. Finally, drug screening data were downloaded from the GDSC website (https://www.cancerrxgene.org/downloads/bulk_download), containing ln(IC_50_) values for drug–cell line pairs. Only the more recently available dataset (GDSC2) was used.

### 2.3 Drug data and feature extraction

A list of all unique drugs of drug–cell line pairs from the GDSC dataset was generated, and their respective Simplified Molecular Input Line Entry System (SMILES) were retrieved from PubChem using PubChemPy (Swain 2014). Unfortunately, SMILES notation was not available for all compounds. Of the 285 compounds from the GDSC2 dataset, only 228 had available SMILES data, which were then subjected to feature extraction to obtain structural information for each drug using two different approaches. The Mordred Python package (Moriwaki *et al.* 2018) was used to retrieve 1D and 2D descriptors for all the drugs, yielding over 1600 features. Similarly, the recently released DrugTax module (Preto *et al.* 2022a) was used to extract features from the SMILES, yielding 163 additional descriptors.

### 2.4 Dataset composition

Except for the scRNA-seq datasets, all other datasets followed a similar preprocessing protocol to prepare them as inputs for the model. First, 10% of the unique cell lines and drugs were randomly selected as part of the validation datasets, hereafter referred to as leave-cell-out and leave-drug-out datasets, respectively. Once the validation cell lines and drugs were extracted, the remaining drug–cell line pairs were further split into training and testing datasets according to a 70–30 train-test split. Features that displayed zero variance were removed as they did not contribute to the prediction. All datasets were finally standardized by first calculating the mean and standard deviation values from the training data and then transforming the datasets based on such values. Because not all datasets from CCLE had the same data available for all cells, there was a minority of missing values that were replaced with zeros, as previously reported (Preto *et al.* 2022b). Substituting absent values with zeros may not consistently be the optimal strategy, primarily because of potential distortions in the data distribution or misinterpretations that can arise. However, in this specific context, zeros precisely reflected the absence of biological entities within our datasets. This makes this approach more suitable than merely excluding significant cell line data and is more biologically accurate than the alternative of imputing values using information from other cell lines. These missing values represented <2% of the values in all datasets, except for the CCLE methylation dataset, which had only 55 of the 66 cell lines available. Moreover, in the GDSC2 drug screening dataset, any repeated drug–cell line pairs with the same ln(IC_50_) values were averaged. To match these datasets with available single-cell data, only cell lines with available scRNA-seq data were selected.

For scRNA-seq datasets, the same set of single cells from each cell line was used for both training and testing datasets. In this case, it was not necessary to split single cells into training and testing datasets because the target variable was not representative of individual cells but rather of the drug–cell line pair. Cell lines that were selected for the leave-cell-out split, on the other hand, were set apart and their scRNA-seq data were not used for training. Given that not all cell lines had identical numbers of single cells, any missing values were replaced with the median value of the expression of that gene for that cell line to ensure that all cell lines were represented by the same number of single cells and to avoid discarding cell lines with fewer available data. In different runs, 10, 25, or 50 cells from each cell line were randomly selected and placed in a new dataset, yielding new datasets containing a single cell of each cell line for training and evaluation. For example, if we selected 10 single cells from each cell line, but a given cell line had only 6 single cells available, the missing values for 4 other cells would be represented by the median expression value from the 6 single cells for each gene.

### 2.5 Algorithm

**Figure 1.**
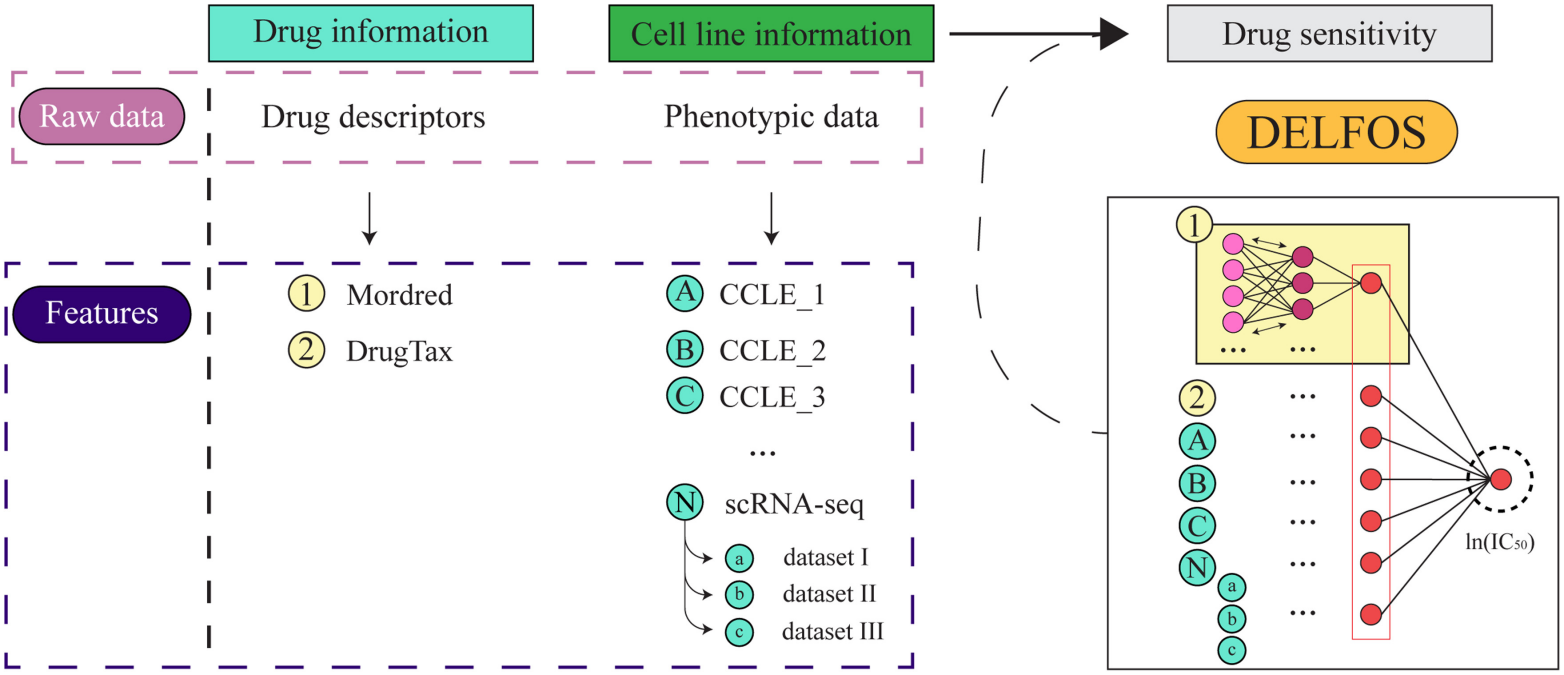
Summary of the proposed architecture and input data for the proposed prediction model, DELFOS.

The proposed model, DELFOS, was developed using the Keras interface in the TensorFlow library (Chollet 2015, Abadi *et al.* 2016). The model was created with a fork-like architecture, in which the datasets were grouped into feature blocks (i.e. drug descriptors, bulk phenotypic data, and scRNA-seq data). Each dataset was processed using its own network, and the output of each network finally converged to yield the output, as illustrated in Fig. 1. The input shape of each dataset was proportional to the number of features in the dataset. All hidden layers of each subnetwork had ReLU as the activation function, except for the output layer of each subnetwork and the overall network, which had a linear activation function. To address potential generalization issues, L1 and L2 regularizers for kernel, bias, and activity were added to all layers except the output layers, and the effects of the presence or absence of dropout layers were evaluated at different dropout rates. The Adam optimizer was used to update the network weights, along with a learning rate of 0.001, using mean squared error (MSE) as the loss function. No specific number of epochs was used for any model because the EarlyStopping callback function of Keras was employed to optimize the training process. The ReduceLROnPlateau Keras callback was used to tune the learning rate.

### 2.6 Benchmark

The optimal hyperparameter configuration for the predictor was tuned with KerasTuner using the Hyperband algorithm (Li *et al.* 2018), both when using scRNA-seq data for prediction and when using the validation loss as a reference.

The DELFOS was evaluated using the following regression metrics: MSE, mean absolute error (MAE), root-mean-square error (RMSE), Pearson correlation coefficient (PCC), Spearman’s rank correlation coefficient (SCC), and *R*^2^. These metrics were also used by Chawla *et al.* to evaluate the performance of eXtreme Gradient Boosting (XGBoost) (Chen and Guestrin 2016) and another Deep Neural Network (DNN) using Precily’s model architecture (Chawla *et al.*, 2022) when trained and tested using our dataset. Accordingly, our datasets were concatenated and normalized prior to training with these models because their architectures were not prepared to receive multiple inputs simultaneously, as in DELFOS. XGBoost had its hyperparameters tuned before being implemented with our datasets. Likewise, our implementation of Precily’s architecture was subjected to hyperparameter optimization with KerasTuner, using the hyperband model prior to training using our datasets. However, the overall DNN architecture as well as the optimization and training protocols proposed by Chawla *et al.* (2022) remained intact.

## 3 Results

### 3.1 Dataset description

Once all datasets were processed and prepared to match the features from drug data, bulk phenotypic data, and scRNA-seq data, 66 cancer cell lines from 16 different tissues and 228 unique drugs were used to train and evaluate model performance (Supplementary Fig. S7). Most cell lines are lung cancer cell lines, whereas some of the other tissues are represented by a single cell line. Uniform Manifold Approximation and Projection analysis revealed that cells belonging to the same cell line clustered together (Supplementary Fig. S8). In total, 52 513 features of 13 575 drug–cell line pairs were used (Supplementary Table S1). The training dataset included 7852 samples, the testing datasets had 3410, the leave-cell-out dataset had 1164, and the leave-drug-out dataset had 1149.

The ln(IC_50_) values provided by the GDSC2 dataset, the target variable for DELFOS, displayed an interquartile range of 3.33 with a median of 3.57 (Supplementary Fig. S9). The mean ln(IC_50_) was 3.14, which was somewhat lower than the median given the presence of many small ln(IC_50_) values. Compounds with very small average ln(IC_50_) values were effective in eliminating cancer cells and were considered powerful anticancer agents, as expected (Supplementary Table S2). In contrast, the compounds with the highest average ln(IC_50_) values were the main antioxidant agents. There were also anticancer agents among the top 10 compounds with increased average values. This is not unexpected given that these compounds are often more effective against specific types of cancer.

### 3.2 Dataset description

Before deploying an ML model, its hyperparameters must be carefully tuned, as they may impact not only its prediction potential, but also its runtime and computational requirements. Different models of DELFOS were optimized using the Hyperband algorithm, which trains a large number of models with different hyperparameters for only a few epochs and, using a tournament bracket-like approach, proceeds only with the best performing models with an increasing number of epochs (Li *et al.* 2018). In both the presence and absence of scRNA-seq data, over 250 different configurations were tested until optimal hyperparameters were established. The complete training hyperparameters are presented in Supplementary Table S3. The different results for the optimal hyperparameter configuration when using scRNA-seq data are not unexpected, as adding more data points will greatly affect the weights and biases by backpropagation. The hyperparameters of the optimized model are listed in Table 2.

**Table 2.** Summary of hyperparameters used in the prediction model using scRNA-seq data.

Optimal hyperparameter settings	
Hyperparameter	Value
Use single cell	TRUE
Hidden layer number	11
Hidden layer size drugs	86
Hidden layer size bulk	100
Hidden layer size single cells	76
Add Dropout layers	FALSE
Learning Rate	0.001
Batch Size	256

### 3.3 Drug sensitivity prediction

#### 3.3.1 DELFOS with no scRNA-seq data

Following hyperparameter optimization and training, the performance of our models was evaluated using the leave-cell-out and leave-drug-out datasets for ln(IC_50_) prediction (Table 3). The performance results for the training datasets are presented in Supplementary Table S4. First, our model, which did not use scRNA-seq data, achieved high prediction performance using the test dataset, with a PCC of 0.90, SCC of 0.86, and an *R*^2^ of 0.80. However, metrics were relatively high, which might be explained by the range of values of the target variable that the model has to predict. For the leave-cell-out dataset, the prediction performance of our model was slightly worse than that of the test dataset, with a PCC of 0.88, an SCC of 0.83, and an *R*^2^ of 0.78. The performance for the leave-drug-out dataset was the worst among all three, as the model was not as effective in predicting ln(IC_50_) for the drug–cell line pairs when presenting the descriptors of an unseen drug. Accordingly, the correlation metrics were not optimal, with a PCC of 0.53, SCC of 0.58, and an *R*^2^ of 0.24. Similarly, the error metrics suggest an increased average deviation between the predicted values and the ground truth.

**Table 3.** Model performance without scRNA-seq data and with the hyperparameters presented in Table 2.

Subset	RMSE	MSE	MAE	Pearson	Spearman	*R* ^2^
Test	1.25	1.57	0.95	0.90	0.86	0.80
Leave-cell-out	1.24	1.53	0.92	0.88	0.83	0.78
Leave-drug-out	2.54	6.43	2.01	0.53	0.58	0.24

#### 3.3.2 DELFOS using scRNA-seq data with the same hyperparameters

Given the slightly worse performance of the previous model when handling unseen data, another model using scRNA-seq but with the same set of hyperparameters was evaluated. The performance results for the test dataset were once again very similar to those of the previous models, both for the correlation and error metrics (Table 4). Accordingly, this model displayed a PCC of 0.90, SCC of 0.86, and *R*^2^ of 0.81, essentially mirroring earlier results. We also ran DELFOS with scRNA-seq using new hyperparameter settings determined by the hyperband algorithm (Supplementary Table S5), which achieved a similar performance (Supplementary Table S6). Likewise, we repeated the implementation using data from 25 or 50 single cells from each cell line instead of only 10, but similar results were obtained (Supplementary Tables S7 and S8 and Supplementary Figs S10 and S11). Thus, these results suggest that scRNA-seq data do not contribute to the final prediction.

**Table 4.** Model performance with scRNA-seq and the hyperparameters presented in Table 2.

Subset	RMSE	MSE	MAE	Pearson	Spearman	*R* ^2^
Test	1.24	1.53	0.93	0.90	0.86	0.81
Leave-cell-out	1.31	1.73	0.99	0.87	0.80	0.76
Leave-drug-out	2.74	7.51	2.15	0.43	0.47	0.19

Following the test dataset, prediction performance was evaluated using the leave-cell-out dataset. We expected an increase in performance, especially when handling unseen cell line data, given additional information. However, DELFOS performed marginally worse than the run without scRNA-seq data when handling data from undetected cell lines. The performance was slightly inferior for both the correlation and error metrics, achieving a PCC of 0.87, an SCC of 0.80, and an *R*^2^ of 0.76. We also evaluated how DELFOS performance was affected by the number of cell lines belonging to a tissue, both in the presence and absence of single-cell data, and found no correlation between the number of cell lines belonging to a tissue and the residuals (Supplementary Figs S12 and S13).

Finally, the prediction performance for the leave-drug-out dataset was similar to that of the first model for all the metrics. Accordingly, the values displayed subtle differences for all metrics, with a PCC of 0.43, SCC of 0.47, and *R*^2^ of 0.19. This was expected because the hyperparameter configuration was identical, and the scRNA-seq data would hardly contribute to how the model handles new drug information. Therefore, the differences, in contrast to the first scenario, are presumably due to chance.

### 3.4 Benchmarking

To understand the performance of DELFOS in relation to other state-of-the-art DRP algorithms, we compared its performance with that of other similar models. None of these models used scRNA-seq data for training; thus, the results of the first run, which did not use scRNA-seq data, were chosen for comparison. The overall characteristics of DELFOS and the different algorithms selected for comparison are shown in Supplementary Table S9. When evaluated using our test dataset, DELFOS outperformed Precily and XGBoost (Table 5).

**Table 5.** Comparison of DELFOS with other algorithms using the same datasets for training and testing.^a^

	Test			Leave-cell-out			Leave-drug-out		
Name	*R* ^2^	PCC	SCC	*R* ^2^	PCC	SCC	*R* ^2^	PCC	SCC
DELFOS	**0.80**	**0.90**	**0.86**	**0.78**	**0.88**	**0.83**	**0.24**	0.53	0.58
*Precily*	0.30	0.60	0.55	0.03	0.57	0.51	0.15	**0.56**	**0.59**
*XGBoost*	0.75	0.87	0.83	0.66	0.82	0.75	0.15	0.49	0.58

a Higher values indicate a better prediction performance in bold.

Our model also outperformed XGBoost and Precily when evaluated using a leave-cell-out dataset. Precily’s results were suboptimal, suggesting that the model could not effectively predict ln(IC_50_) for the unseen cell lines. Similarly, DELFOS performed better than XGBoost when presented with unseen drug data. In contrast, Precily displayed better results than DELFOS for PCC and SCC but not for *R*^2^. This suggests that Precily generated predictions with stronger monotonic correlations with the ground truth values, although these values did not match the data or predictions by DELFOS. Nonetheless, none of these models, including DELFOS, could optimally predict ln(IC_50_) when unseen drug data were presented, highlighting a problem to be addressed in future DRP approaches.

Next, we compared our performance results with those of three other models without using our data. Given the differences in the backbone between models, as well as the available information for their adequate implementation using our data, we simply compared our performance results with the highest reported metrics in the original studies of other DRP models. Overall, DELFOS did not yield the best results among the four models, with two Graph Neural Network-based models, GraphDRP (Nguyen *et al.* 2022) and DeepCDR (Liu *et al.* 2020), slightly outperforming our model in some metrics. However, these models did not report an *R*^2^ value, and thus, the only conclusion that can be reached is that their predicted results are more correlated with the ground truth in monotonic terms. In contrast, DELFOS outperformed both NeRD (Cheng *et al.* 2022) and Precily on the original dataset (Chawla *et al.* 2022) for all metrics.

Regarding the leave-cell-out and leave-drug-out datasets, only a few metrics were presented in the original studies of these models (Table 6). Except for NeRD, which also presented its SCC value, GraphDRP and DeepCDR only reported PCC values. With respect to the available metrics for the leave-cell-out dataset, DELFOS outperformed the other algorithms and matched the DeepCDR performance. However, when presented with unseen drug data, our model outperformed the others on all metrics, although our model was the only one to report *R*^2^. Moreover, DELFOS performed well in relation to the other algorithms in terms of processing time, displaying one of the fastest processing times during model training when compared to other models running their own datasets with default settings (Supplementary Table S10).

**Table 6.** Comparison of the highest reported metrics between DELFOS and other algorithms.^a^

	Test			Leave-cell-out			Leave-drug-out		
Name	*R* ^2^	PCC	SCC	*R* ^2^	PCC	SCC	*R* ^2^	PCC	SCC
DELFOS	**0.80**	0.90	0.86	**0.78**	0.88	**0.83**	**0.24**	**0.53**	**0.58**
*NeRD*	0.75	0.87	0.84		0.84	0.81		0.37	0.29
*Precily*	0.77	0.88							
*GraphDRP*		**0.93**			0.85			0.32	
*DeepCDR*		0.92	**0.90**		**0.89**			0.50	

a Higher values indicate a better prediction performance in bold.

## 4 Discussion

Our results indicate that DELFOS can efficiently predict ln(IC_50_) values for drug–cell line pairs, even for cell lines that have never interacted before. DELFOS matched or outperformed several recently published DRP algorithms trained using data from GDSC and CCLE. We expected that including scRNA-seq data in the pipeline would improve the prediction performance, particularly for underrepresented tissues. However, we observed no significant benefit when incorporating these data, at least when using this approach. Owing to high variability and noise, scRNA-seq data may yield no patterns with sufficient correlation to drug response to improve the overall prediction performance. This also suggests that the model could receive too much cell data and become overfitted to cell features, which can be amplified when scRNA-seq is provided. More data are required to identify better alternatives for preparing single-cell data for ML. Missing values may also contribute to this, particularly in the CCLE methylation dataset, which displays a relatively high number of missing values. Moreover, our model suffers from low performance when receiving data from unseen compounds. This was also observed by other authors (Li *et al.* 2021, Cheng *et al.* 2022, Nguyen *et al.* 2022), even when molecular descriptors are not used to characterize drugs, highlighting a problem that should be addressed in the future. Finding proper strategies to link drug targets and pathway data as features, as well as better ways to integrate single-cell data, could be beneficial to the performance of future algorithms.

Although many other models are available, Precily’s model was chosen for comparison with our model because (i) it was very recently published and (ii) it follows a streamlined approach that had good performance in its original study (Chawla *et al.* 2022). Likewise, XGBoost was also chosen for comparison given its scalability, popularity, and overall structure, because it is a decision tree-based method rather than an ANN-based method, and thus could provide interesting insights. The performance of the former was much lower than that of its original study, most likely because of the large amount of data used in our dataset in contrast to that of the original study. Outperforming XGBoost was also unexpected, as it is often considered a strong option when using tabular data, and some have even proposed an XGBoost-DL ensemble as a powerful alternative (Shwartz-Ziv and Armon 2022). Most studies do not completely report their feature extraction and selection approaches nor provide insights into the specific hyperparameters required for training or optimization, making it difficult to implement other models using our data and highlighting the need for clearer replicability guidelines in computational biology. It is unfair for our model to simply use the metrics of other studies as a means for comparison, given the different input data types and feature processing protocols between models. Likewise, it would be unfair to run our datasets simply using other models, given that the feature extraction process and optimization might differ. For example, while DeepCDR (Liu *et al.* 2020) and GraphDRP (Nguyen *et al.* 2022) reported relatively high performance in their own studies, as shown in Table 6, these models performed much worse when benchmarked for comparison with NeRD (Cheng *et al.* 2022), with *R*^2^ values of 0.57 and 0.72, respectively. Therefore, direct comparisons should be carefully considered.

Overall, DELFOS performed quite well compared to recent algorithms and could leverage multi-omics data for the prediction of drug responses in several cancer cell lines.

## Supplementary Material

btad645_Supplementary_Data

## Data Availability

All datasets used in this study are publicly available and have been published in other papers. The scRNA-seq datasets used in the development of DELFOS are referenced in the manuscript. All datasets as well as scripts used for analysis and drug response prediction are available at github.com/MoreiraLAB/delfos.
