# Supplementary material for: DELFOS—drug efficacy leveraging forked and specialized networks—benchmarking scRNA-seq data in multi-omics-based prediction of cancer sensitivity
Source: Bioinformatics. 2023 Oct 20;39(11):btad645. doi: 10.1093/bioinformatics/btad645 (PMC10627353; doi:10.1093/bioinformatics/btad645)
Supplement: btad645_Supplementary_Data [file btad645_supplementary_data.docx]

Systems biology

**DELFOS - Drug Efficacy Leveraging Forked and Specialized Networks - Benchmarking scRNA-seq data in multi-omics-based prediction of cancer sensitivity**

Luiz Felipe Piochi^1,2,3^, António J Preto^2,3,4^, Irina S Moreira^1,2,3,*^

^1^ Department of Life Sciences, University of Coimbra, Calçada Martim de Freitas, 3000-456 Coimbra, Portugal

^2^ CNC - Center for Neuroscience and Cell Biology, Center for Innovative Biomedicine and Biotechnology, University of Coimbra, Coimbra, Portugal

^3^ CIBB - Center for Innovative Biomedicine and Biotechnology, 3004-504 Coimbra, Portugal

^4^ PhD Programme in Experimental Biology and Biomedicine, Institute for Interdisciplinary Research (IIIUC), University of Coimbra, Casa Costa Alemão, 3030-789 Coimbra, Portugal

*To whom correspondence should be addressed.

# Supplementary Information

# Supplementary Tables

Table S1. Summary of the number of features per dataset.

| Dataset | Number of features |
| --- | --- |
| Mordred | 700 |
| DrugTax | 45 |
| CCLE_chromatin | 45 |
| CCLE_copynumber | 16,386 |
| CCLE_expression | 16,246 |
| CCLE_methylation | 16,353 |
| CCLE_miRNA | 738 |
| scRNA-seq | 2,000 |
| Total | 52,513 |

Table S2. Summary of the compounds with the top ten lowest and highest mean ln(IC50) values in our target variable dataset.

| Drugs with ten lowest mean ln(IC_50_) values | | | Drugs with ten highest mean ln(IC_50_) values | | |
| --- | --- | --- | --- | --- | --- |
| Drug name | **Mean ln(IC_50_)** | **Class** | **Drug name** | **Mean ln(IC_50_)** | **Class** |
| Romidepsin | -4.81 | histone deacetylase inhibitor | L-ascorbate | 10.65 | antioxidant |
| Bortezomib | -4.43 | proteasome inhibitor | N-acetyl cysteine | 10.23 | antioxidant |
| Sepantronium bromide | -3.80 | proapoptotic agent | Glutathione | 9.49 | antioxidant |
| Daporinad | -3.64 | antiangiogenic | Alpha-lipoic acid | 8.20 | antioxidant |
| Dactinomycin | -3.28 | antibiotic | Temozolomide | 6.99 | alkylating agent |
| Docetaxel | -2.76 | mitosis inhibitor | Nelarabine | 6.52 | DNA synthesis inhibitor |
| Vinblastine | -2.59 | microtubule formation inhibitor | EPZ5676 | 6.13 | histone methyltransferase inhibitor |
| Staurosporine | -2.58 | protein kinases inhibitor | GSK2830371 | 5.99 | phosphatase inhibitor |
| Vinorelbine | -2.20 | mitosis inhibitor | MIRA-1 | 5.97 | proapoptotic agent |
| Dinaciclib | -2.00 | kinase inhibitor | Motesanib | 5.94 | tyrosine kinase inhibitor |

Table S3. Hyperparameters used for DELFOS optimization using callbacks EarlyStopping and ReduceLROnPlateau with the objective of optimizing the validation loss.

| Hyperparameter | Range | Step |
| --- | --- | --- |
| hidden_layer_number | [3, 14] | 1 |
| hidden_layer_size_cells | [20, 250] | 4 |
| hidden_layer_size_drugs | [20, 250] | 4 |
| add_dropout_cells | (True, False) | choice |
| add_dropout_drugs | (True, False) | choice |
| add_dropout_single | (True, False) | choice |
| dropout_rate_cells | [0.1, 0.6] | 0.1 |
| dropout_rate_drugs | [0.1, 0.6] | 0.1 |
| dropout_rate_single | [0.1, 0.6] | 0.1 |
| use_single_cell | (True, False) | choice |
| learning_rate | (1e-2, 1e-3, 1e-4, 1e-5) | choice |
| batch_size | (32, 64, 128, 256) | choice |
| epochs | 200 | - |

Table S4. Performance results of DELFOS for the training datasets with and without using scRNA-seq data.

| Model | RMSE | MSE | MAE | Pearson | Spearman | R^2^ |
| --- | --- | --- | --- | --- | --- | --- |
| w/o scRNA-seq | 1.18 | 1.40 | 0.89 | 0.91 | 0.88 | 0.82 |
| w/ scRNA-seq | 1.19 | 1.41 | 0.89 | 0.91 | 0.88 | 0.82 |

Table S5. Optimal hyperparameters were determined specifically for DELFOS using the scRNA-seq data.

| Hyperparameter | Value |
| --- | --- |
| Use single cell | TRUE |
| Hidden layer number | 11 |
| Hidden layer size drugs | 64 |
| Hidden layer size bulk | 48 |
| Hidden layer size single cells | 76 |
| Add Dropout layers | FALSE |
| Learning Rate | 0.001 |
| Batch Size | 256 |

Table S6. Performance results of model with new hyperparameter settings using scRNA-seq data.

| Subset | RMSE | MSE | MAE | Pearson | Spearman | R^2^ |
| --- | --- | --- | --- | --- | --- | --- |
| Test | 1.24 | 1.54 | 0.94 | 0.90 | 0.86 | 0.80 |
| Leave-cell-out | 1.34 | 1.81 | 1.00 | 0.88 | 0.81 | 0.74 |
| Leave-drug-out | 2.81 | 7.87 | 2.23 | 0.43 | 0.51 | 0.07 |

Table S7. Performance results of model using scRNA-seq data from 25 single cells of each cell line.

| Subset | RMSE | MSE | MAE | Pearson | Spearman | R^2^ |
| --- | --- | --- | --- | --- | --- | --- |
| Test | 1.29 | 1.66 | 0.98 | 0.90 | 0.86 | 0.79 |
| Leave-cell-out | 1.26 | 1.58 | 0.93 | 0.88 | 0.82 | 0.77 |
| Leave-drug-out | 2.74 | 7.51 | 2.15 | 0.43 | 0.47 | 0.12 |

Table S8. Performance results of model using scRNA-seq data from 50 single cells of each cell line.

| Subset | RMSE | MSE | MAE | Pearson | Spearman | R^2^ |
| --- | --- | --- | --- | --- | --- | --- |
| Test | 1.23 | 1.52 | 0.93 | 0.90 | 0.86 | 0.81 |
| Leave-cell-out | 1.27 | 1.61 | 0.95 | 0.88 | 0.81 | 0.77 |
| Leave-drug-out | 2.74 | 7.49 | 2.16 | 0.44 | 0.47 | 0.12 |

Table S9. Overall characteristics of DELFOS and the additional algorithms selected for comparison. Abbreviations: CHR: Chromatin accessibility, CNN: Convolutional Neural Network, CNV: Copy Number Variation, CV: Cross-Validation, DNN: Deep Neural Network, DRUG: Drug structure, EXP: Expression, GNN: Graph Neural Network, MET: Methylation, miRNA: MicroRNA, MUT: Mutation, Sen-Res: Sensitive-Resistant, scEXP, single-cell expression.

| **NAME** | Method | Datasets | Input Omics | Validation Strategy | Target |
| --- | --- | --- | --- | --- | --- |
| DELFOS | DNN | GDSC, CCLE | scEXP, EXP, CHR, miRNA, CNV, MET, DRUG | 70-30 train-test split, leave-some-out CV | IC_50_ |
| *NeRD* (Cheng *et al.* 2022) | CNN/GNN | GDSC, CCLE | CNV, DRUG, miRNA | 5-fold CV, 80-10-10 train-test-validation split | IC_50_ |
| *Precily* (Chawla *et al.* 2022) | DNN | GDSC, CCLE, TCGA | EXP, DRUG | 90-10 train-test split, 5-fold CV | IC_50_ / Sen-Res |
| *GraphDRP* (Nguyen *et al.* 2022) | GNN | GDSC, CCLE | EXP, CNV, MUT, DRUG | 80-10-10 train-test-validation split | IC_50_ |
| *DeepCDR* (Liu *et al.* 2020) | GNN | GDSC, CCLE, TCGA | EXP, CNV, MET, DRUG | 80-20 train-test split, leave-one-out CV | IC_50_ / Sen-Res |

Table S10. Comparison of CPU and Wall Clock times during training between models used in benchmarks. All measurements were retrieved from a six-core 3.8 GHz, 16GB RAM machine.

| NAME | Avg. CPU time per training epoch | Avg. Wall Clock time per training epoch | Total training CPU time | Total training Wall Clock time |
| --- | --- | --- | --- | --- |
| DELFOS | 7.64s | 4.05s | 449.63s | 260.45s |
| DELFOS + scRNA-seq data | 8.72s | 13.25s | 1807.63s / 0.50h | 2914.01s / 0.81h |
| *NeRD* (Cheng *et al.* 2022) | 988.69s | 197.95s | 93.92h | 16.49h |
| *Precily* (Chawla *et al.* 2022) | 6.74s | 32.67s | 420.84s | 696.91s |
| *GraphDRP* (Nguyen *et al.* 2022) | 829.03s | 170.83s | 79.44h | 14.24h |
| *DeepCDR* (Liu *et al.* 2020) | 8332.23s | 2217.76s | 266.17h | 70.85h |

# Supplementary Figures


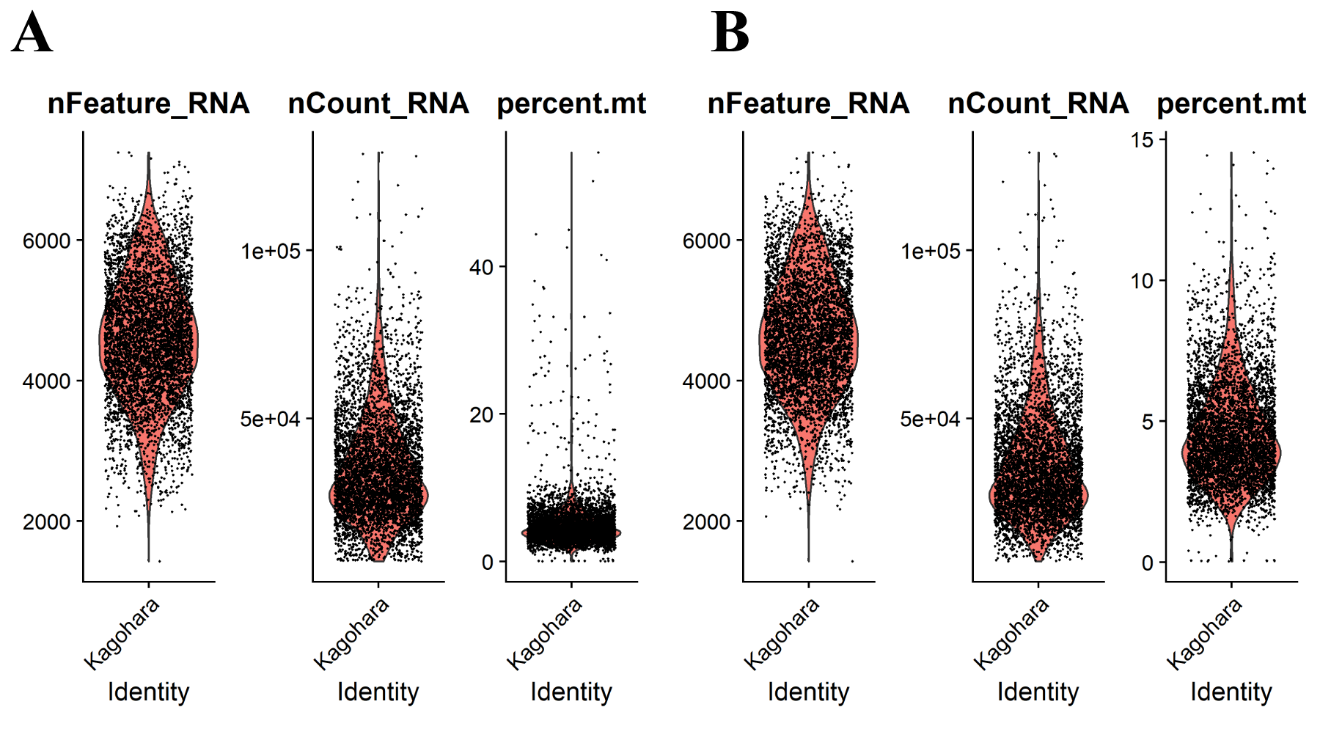


Figure S1. Violin plots comparing cells belonging to Kagohara et al. (Kagohara et al. 2020) dataset before (A) and after (B) filtering out cells expressing over 15% of mitochondrial genes.


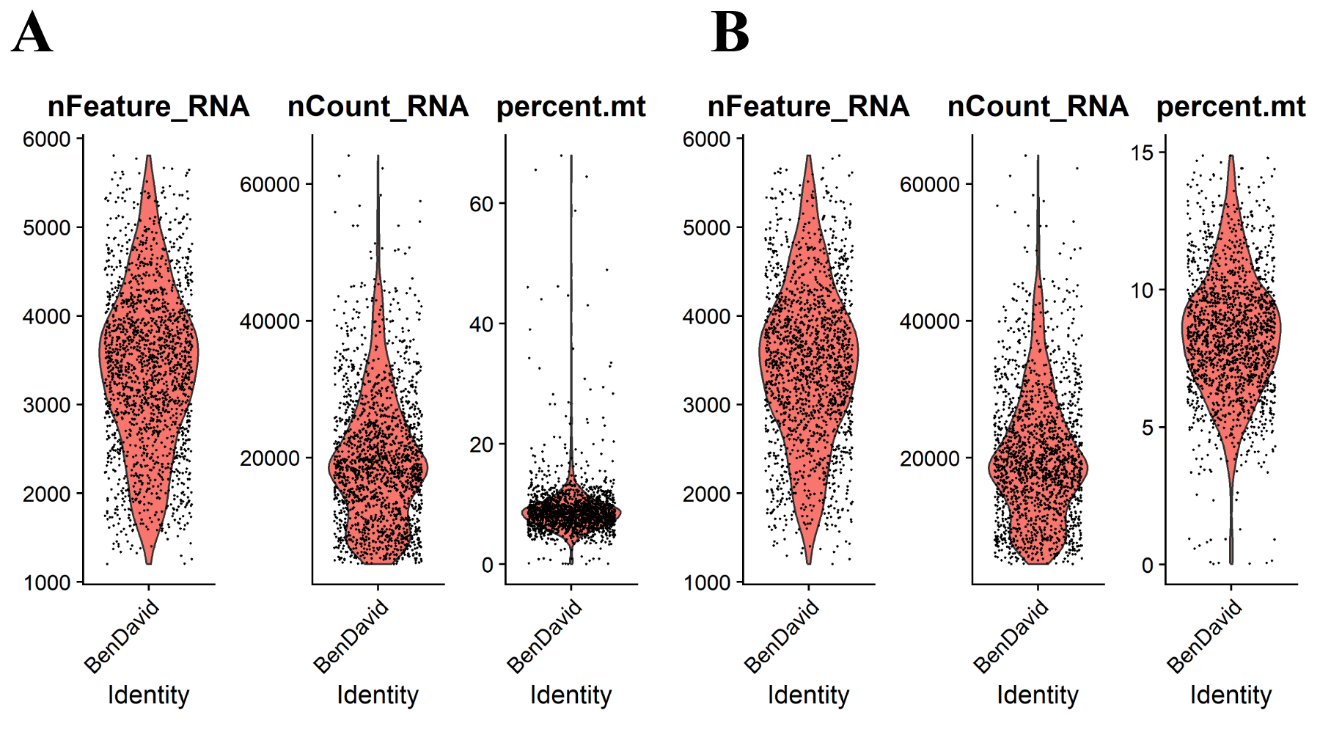


Figure S2. Violin plots comparing cells belonging to Ben-David et al. (Ben-David et al. 2018) dataset before (A) and after (B) filtering out cells expressing over 15% of mitochondrial genes*.*


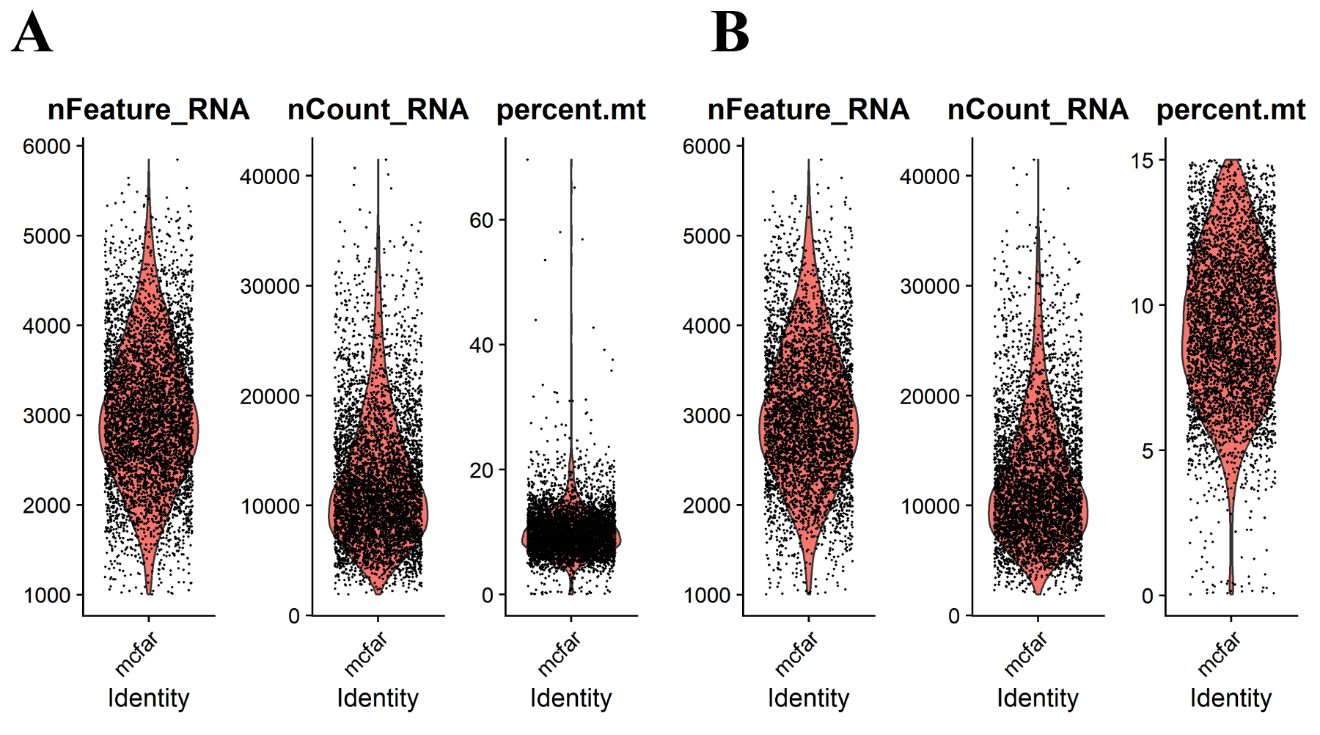


Figure S3. Violin plots comparing cells belonging to McFarland et al. (McFarland et al. 2020) dataset before (A) and after (B) filtering out cells expressing over 15% of mitochondrial genes.


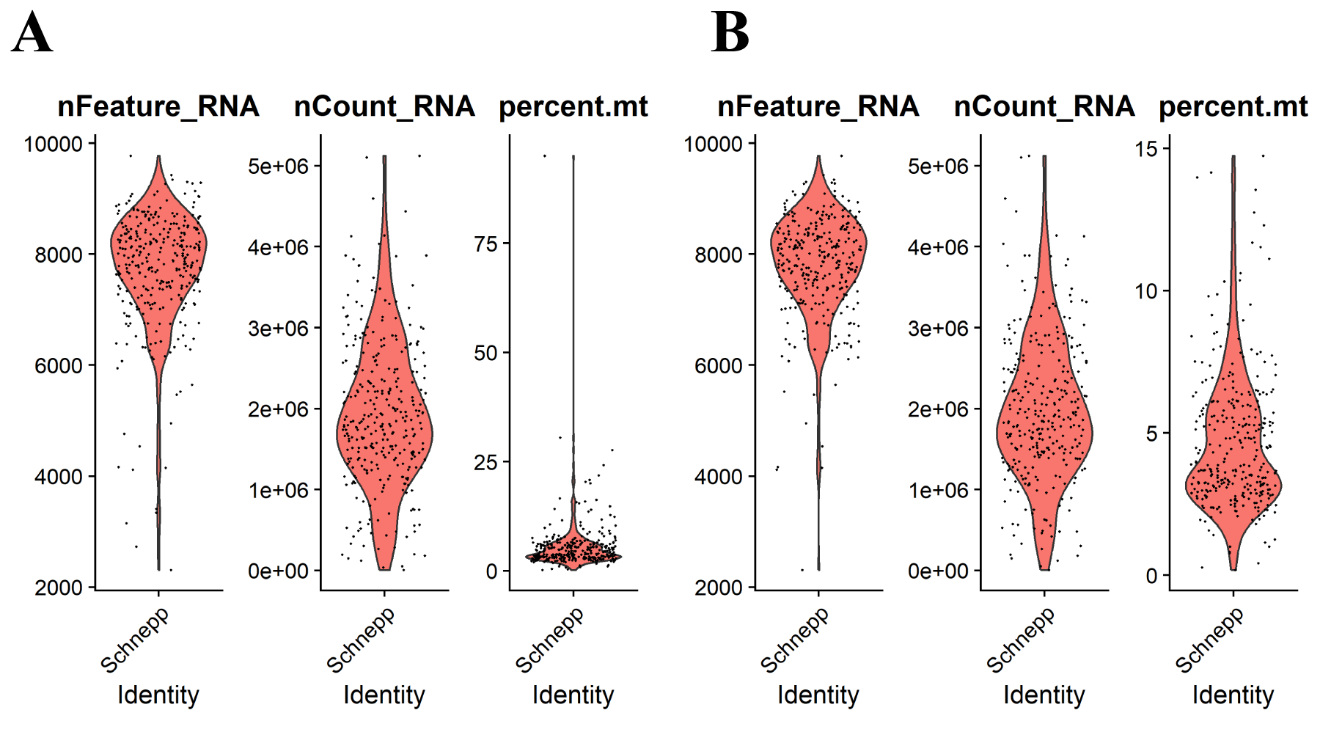


Figure S4. Violin plots comparing cells belonging to Schnepp et al. (Schnepp et al. 2020) dataset before (A) and after (B) filtering out cells expressing over 15% of mitochondrial genes.


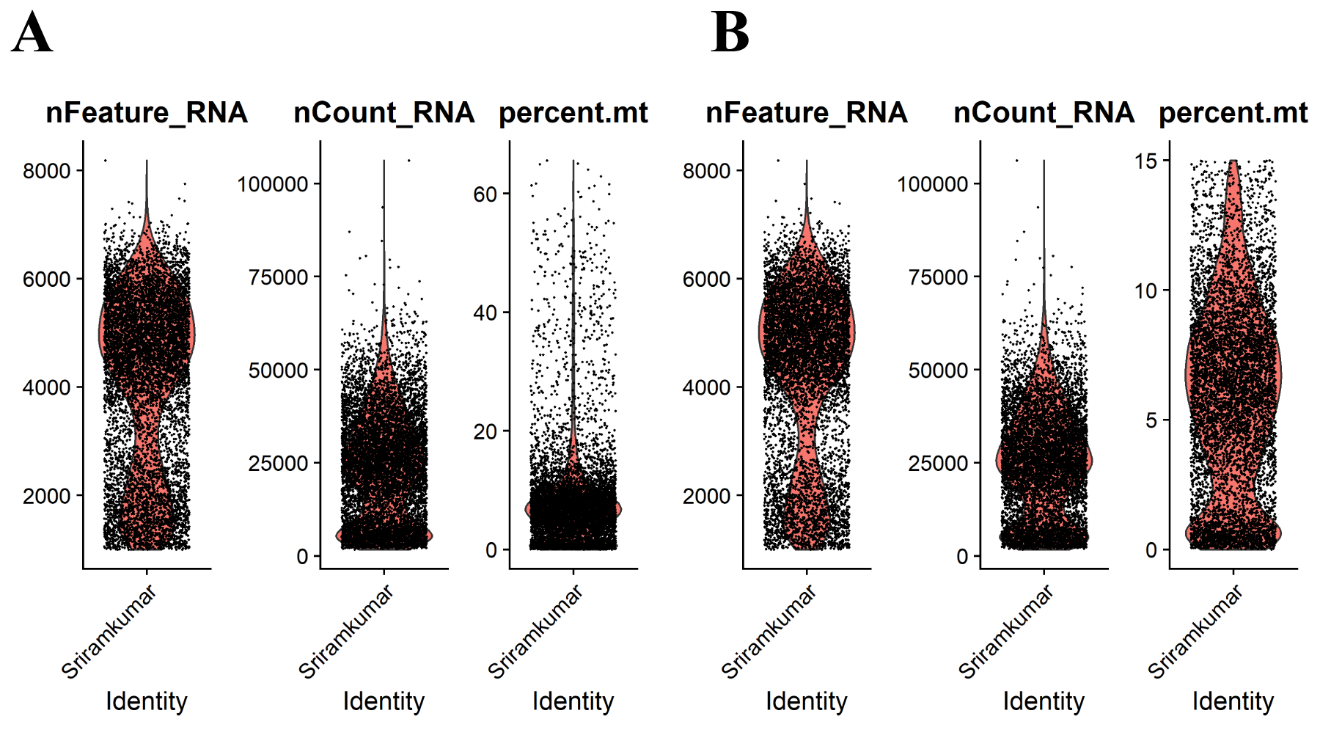


Figure S5. Violin plots comparing cells belonging to Sriramkumar et al. (Sriramkumar et al. 2022) dataset before (A) and after (B) filtering out cells expressing over 15% of mitochondrial genes.


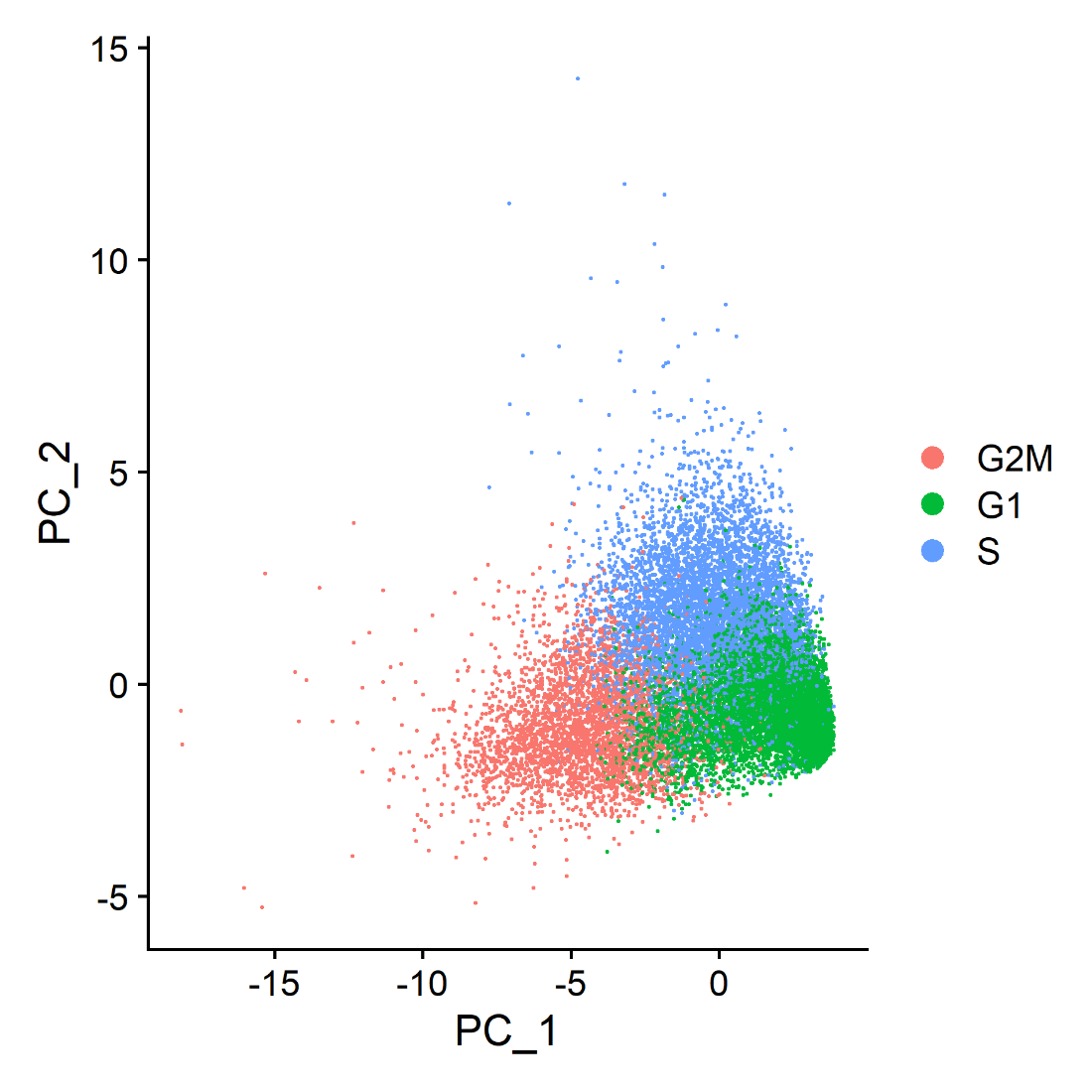


Figure S6. PCA plot displaying the distribution of cells in our scRNA-seq dataset colored according to the cell cycle stage in which the data were retrieved.


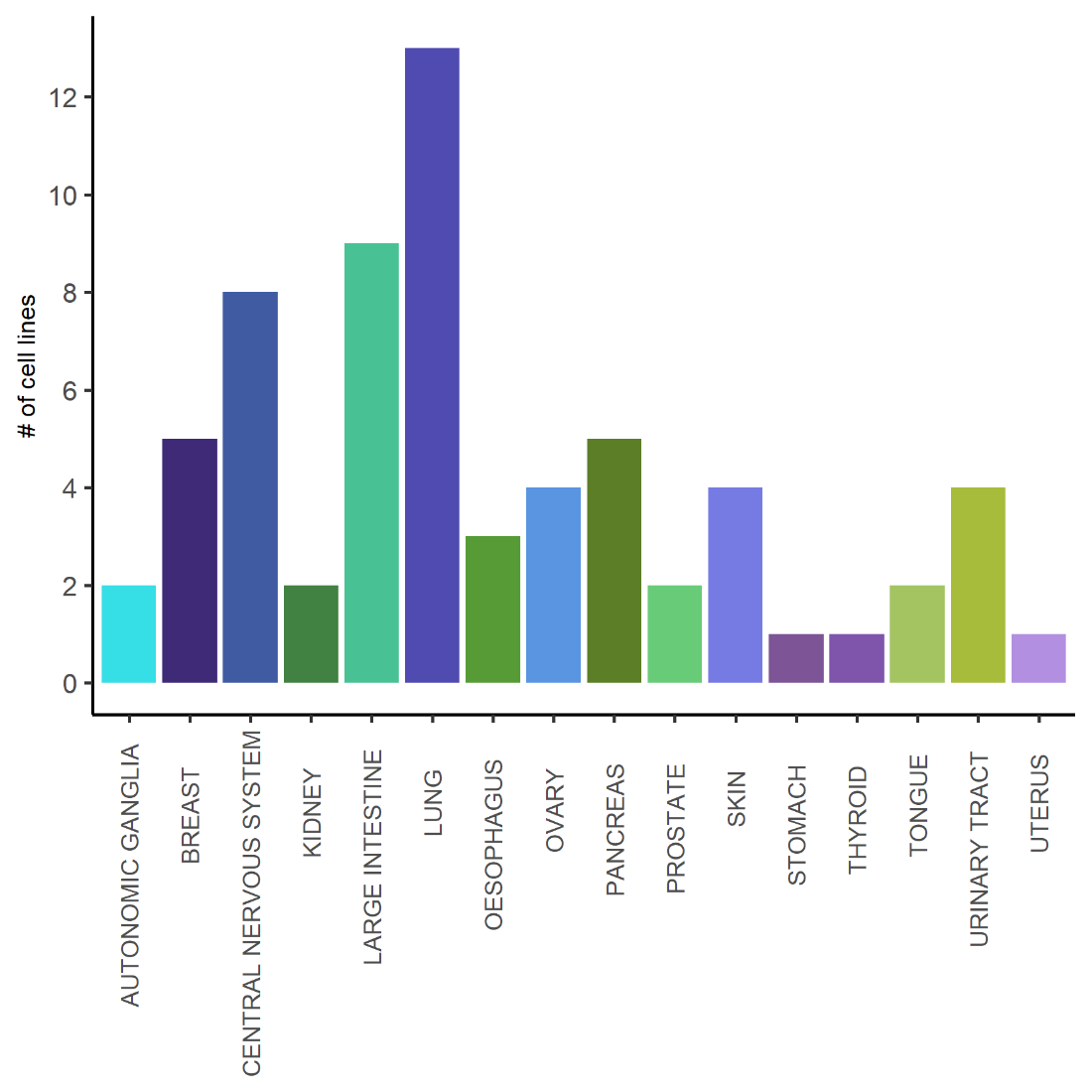


Figure S7. Distribution of the 66 cell lines across 16 tissues in the used datasets.


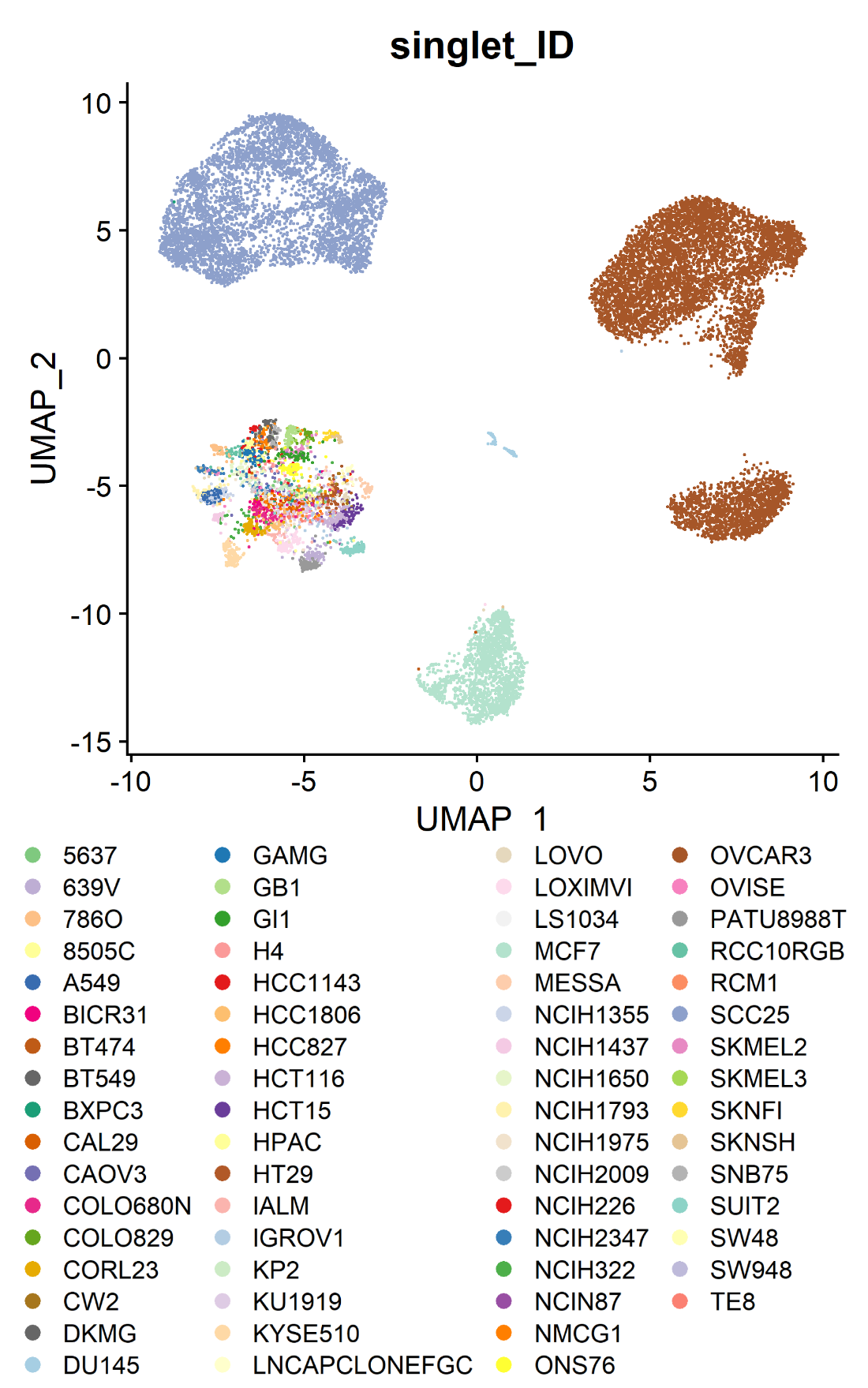


Figure S8. The UMAP plot displays the distribution between different cell types, with cells belonging to the same cell line grouped by color, as shown in the legend below. Larger clusters represent the cells derived from different datasets.


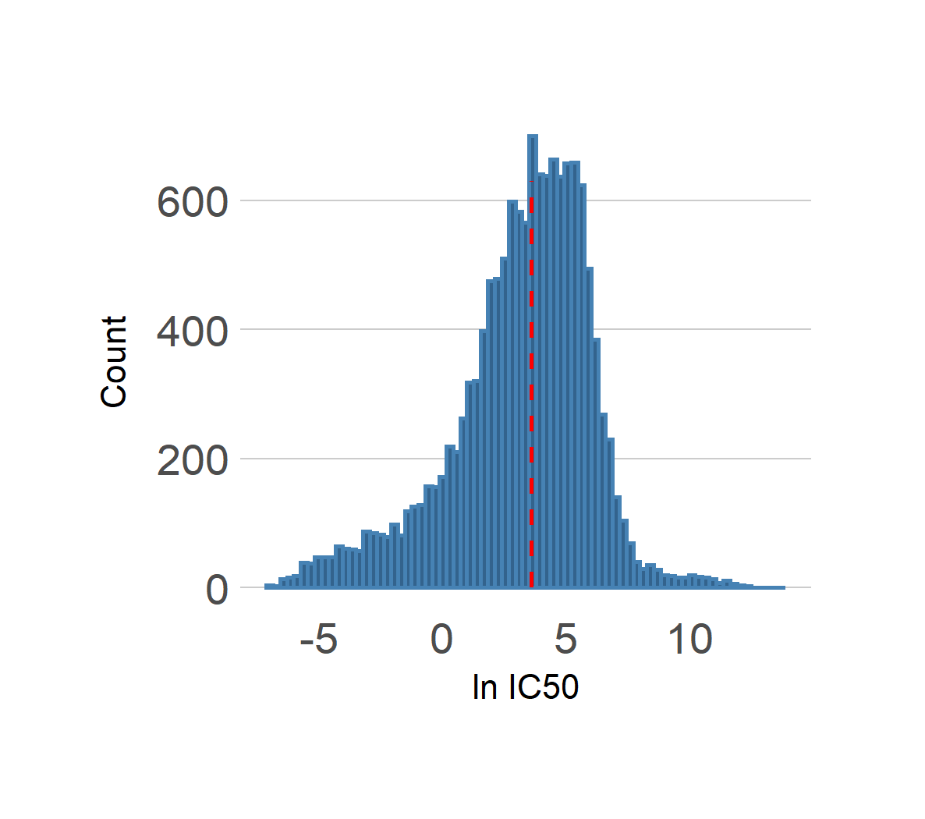


Figure S9. Histogram of the target variable ln(IC_50_) in the GDSC2 dataset. The red lines represent the median values. The number of bins was estimated using the Freedman-Diaconis rule.


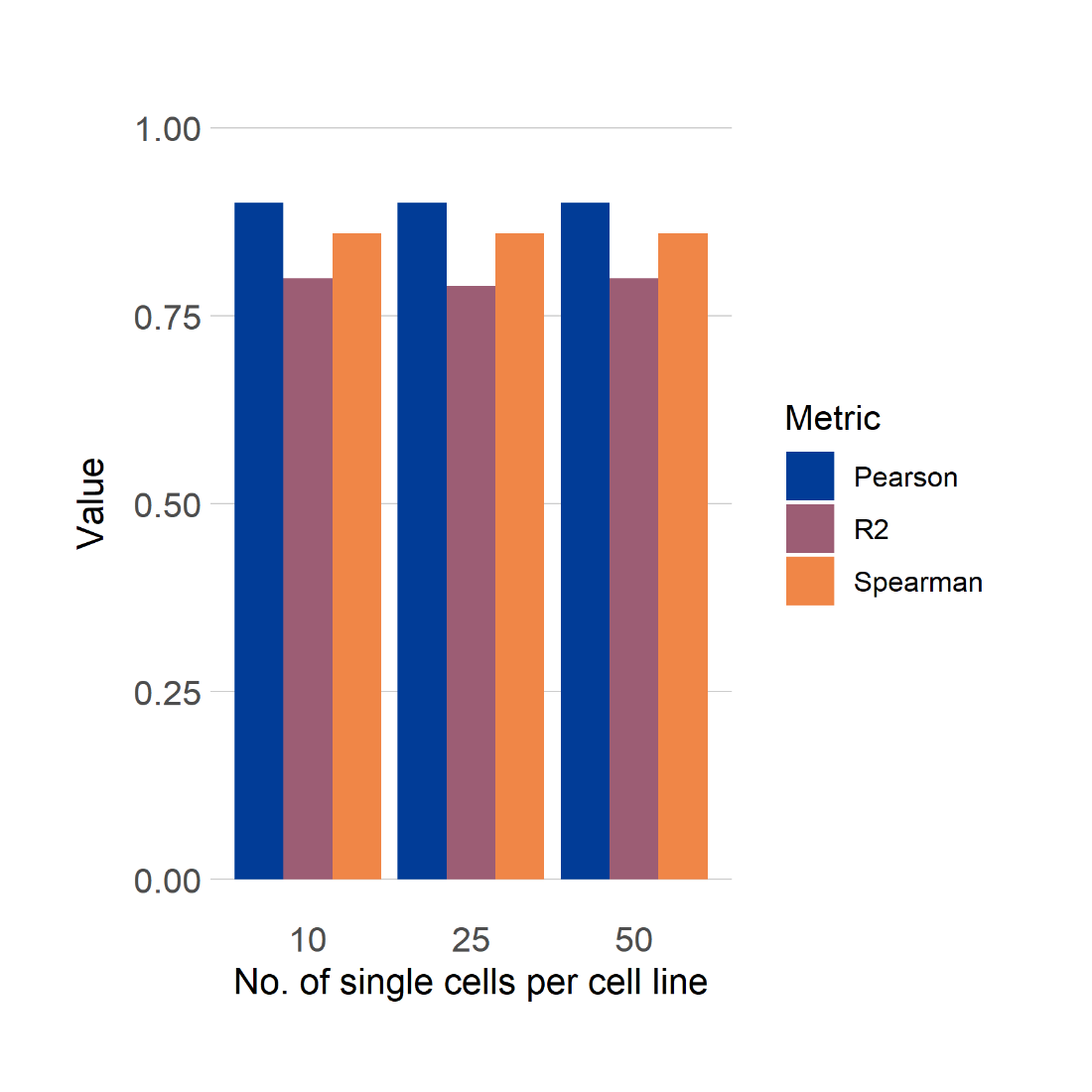


Figure S10. Bar plot comparing the performance values of different correlation metrics using scRNA-seq data from different numbers of single cells per cell line. Higher values indicate better performance.


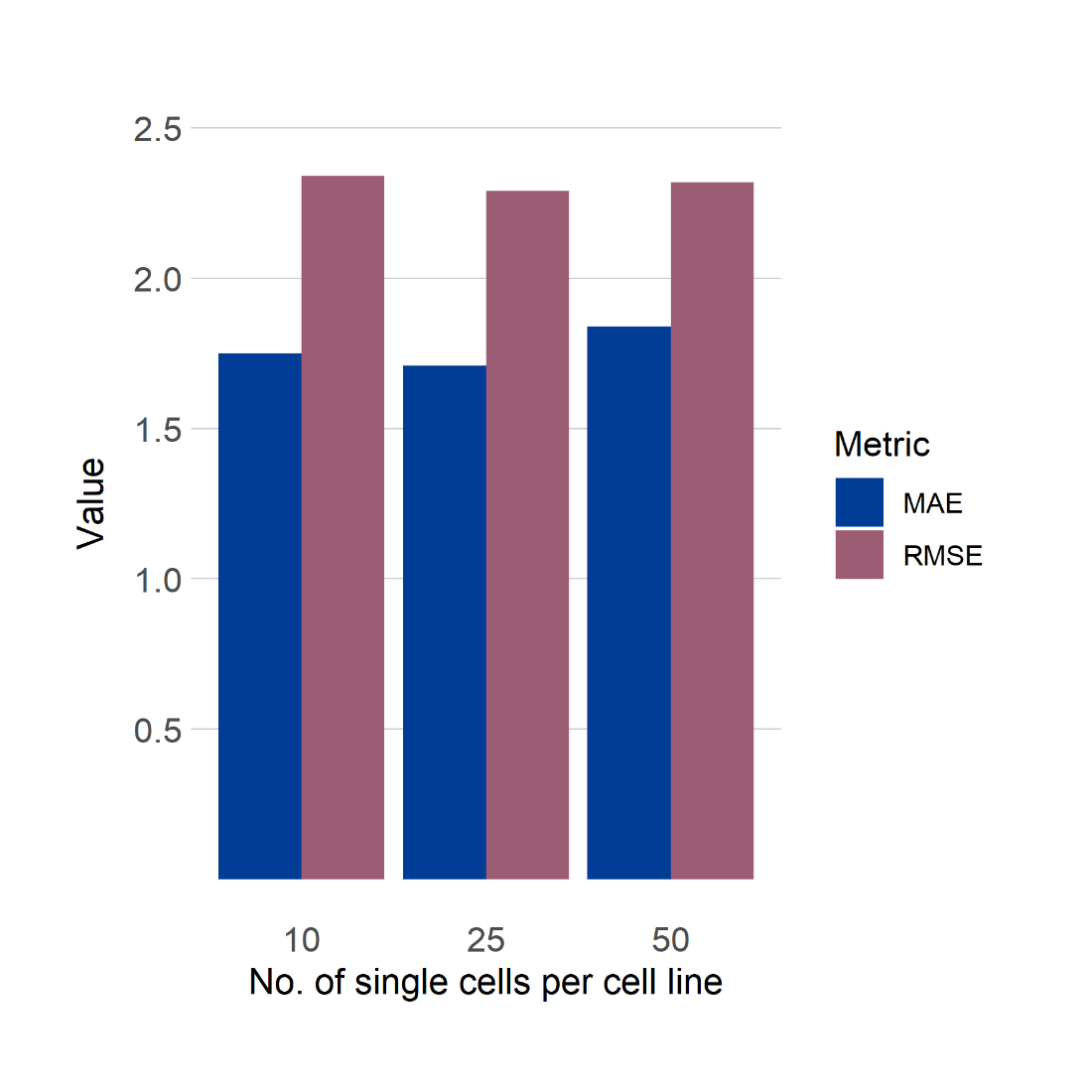


Figure S11. Bar plot comparing the performance values of error metrics using scRNA-seq data from different numbers of single cells per cell line. Smaller values indicate better performance.


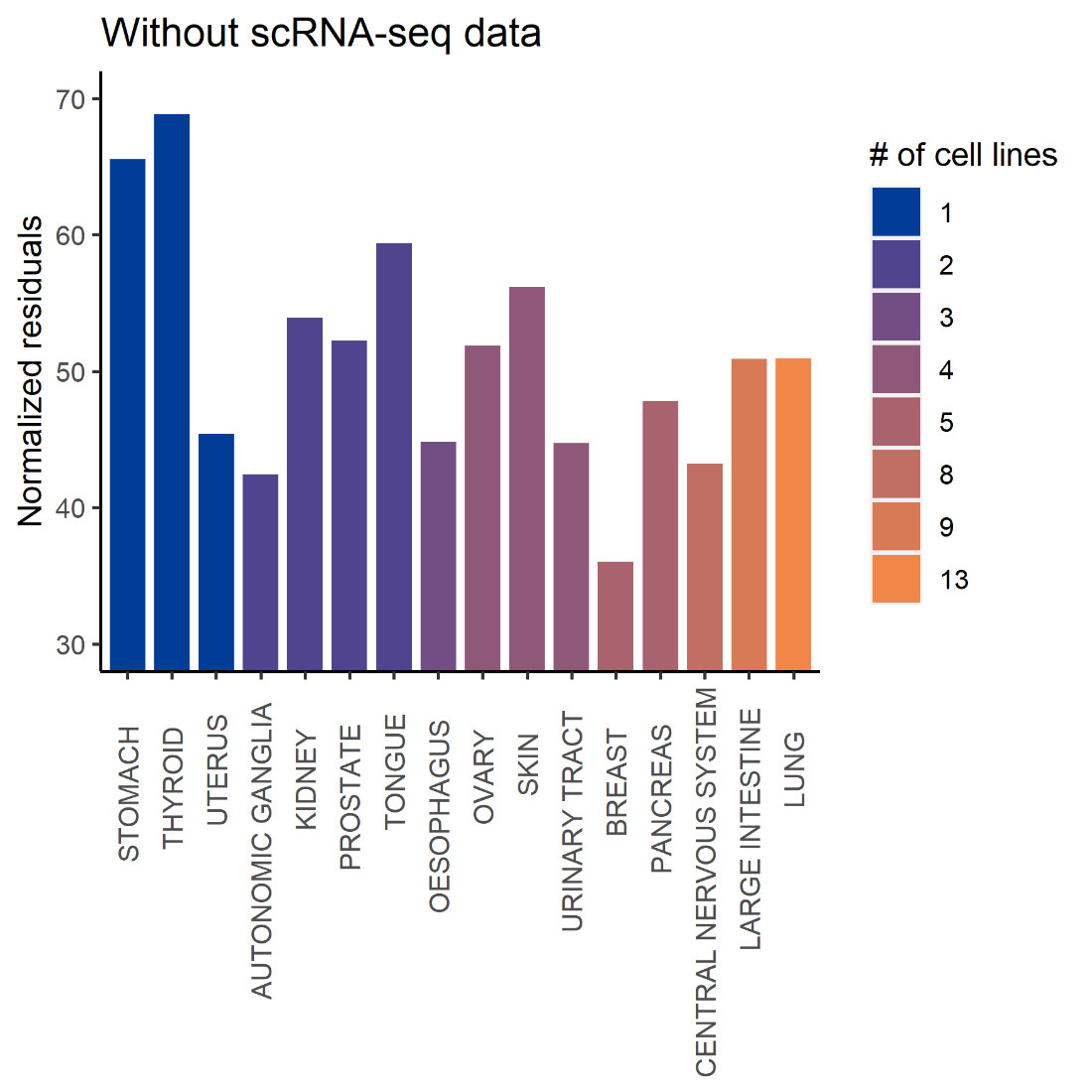


Figure S12. Bar plot correlating the sum of residuals normalized by the number of cell lines belonging to a tissue without using scRNA-seq data. There was no correlation between the number of cell lines and the residuals (PCC = 0.02, SCC = 0.03).


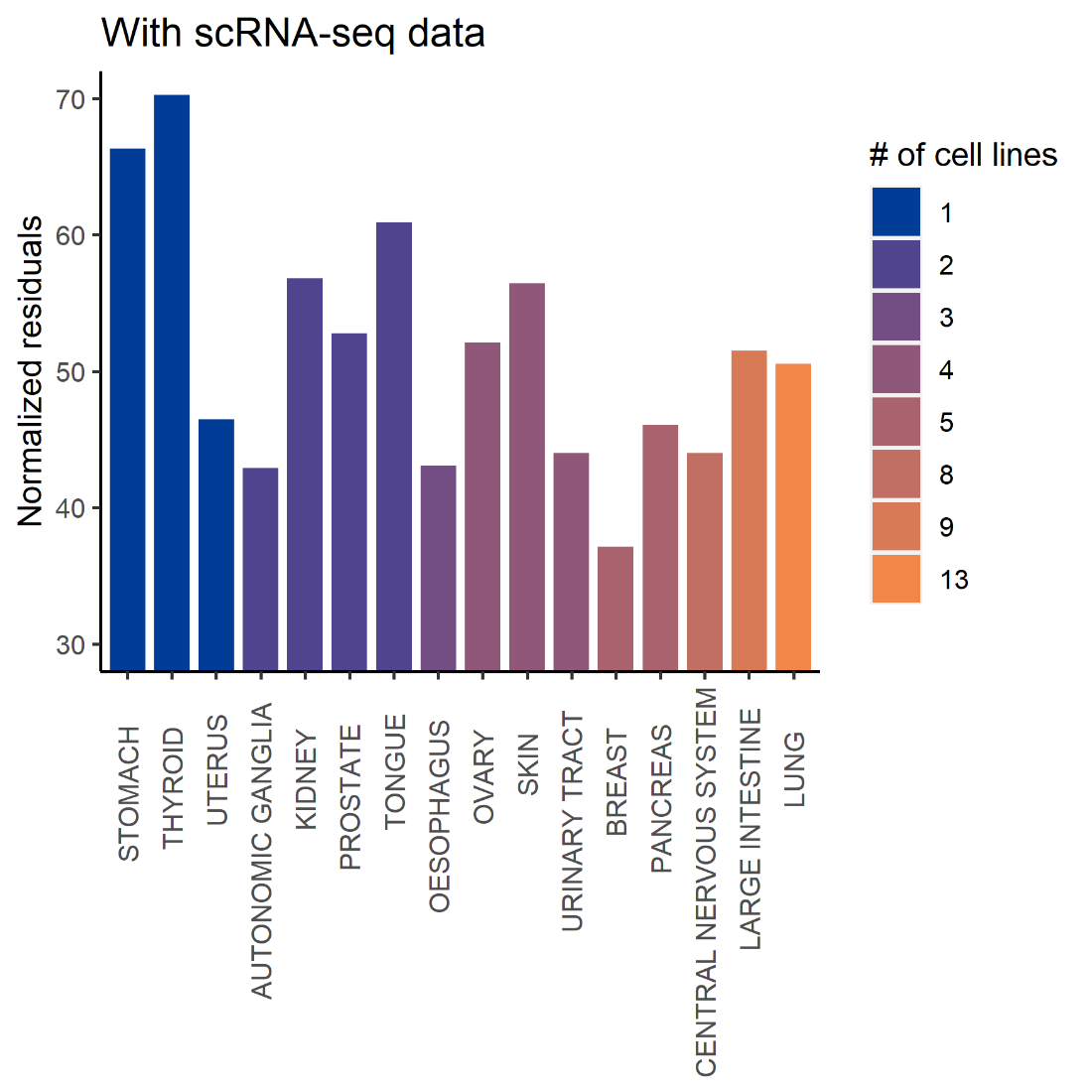


Figure S13. Bar plot correlating the sum of residuals normalized by the number of cell lines belonging to a tissue when using scRNA-seq data. There was no correlation between the number of cell lines and the residuals (PCC = 0.01, SCC = 0.02).

Ben-David U, Siranosian B, Ha G *et al.* Genetic and transcriptional evolution alters cancer cell line drug response. *Nature* 2018;**560**:325–30.

Chawla S, Rockstroh A, Lehman M *et al.* Gene expression based inference of cancer drug sensitivity. *Nat Commun* 2022;**13**, DOI: 10.1038/s41467-022-33291-z.

Cheng X, Dai C, Wen Y *et al.* NeRD: a multichannel neural network to predict cellular response of drugs by integrating multidimensional data. *BMC Med* 2022;**20**:1–16.

Kagohara LT, Zamuner F, Davis-Marcisak EF *et al.* Integrated single-cell and bulk gene expression and ATAC-seq reveals heterogeneity and early changes in pathways associated with resistance to cetuximab in HNSCC-sensitive cell lines. *Br J Cancer* 2020;**123**:101–13.

Liu Q, Hu Z, Jiang R *et al.* DeepCDR: A hybrid graph convolutional network for predicting cancer drug response. *Bioinformatics* 2020;**36**:I911–8.

McFarland JM, Paolella BR, Warren A *et al.* Multiplexed single-cell transcriptional response profiling to define cancer vulnerabilities and therapeutic mechanism of action. *Nat Commun* 2020;**11**, DOI: 10.1038/s41467-020-17440-w.

Nguyen T, Nguyen GTT, Nguyen T *et al.* Graph Convolutional Networks for Drug Response Prediction. *IEEE/ACM Trans Comput Biol Bioinform* 2022;**19**:146–54.

Schnepp PM, Shelley G, Dai J *et al.* Single-Cell Transcriptomics Analysis Identifies Nuclear Protein 1 as a Regulator of Docetaxel Resistance in Prostate Cancer Cells. *Mol Cancer Res* 2020;**18**:1290–301.

Sriramkumar S, Metcalfe TX, Lai T *et al.* Single-cell analysis of a high-grade serous ovarian cancer cell line reveals transcriptomic changes and cell subpopulations sensitive to epigenetic combination treatment. 2022:1–15.
